# Lower estimated glomerular filtration rates in patients on long term lithium: a comparative study and a meta-analysis of literature

**DOI:** 10.1186/1471-244X-14-4

**Published:** 2014-01-08

**Authors:** Chaturaka Rodrigo, Nipun Lakshitha de Silva, Ravindi Gunaratne, Senaka Rajapakse, Varuni Asanka De Silva, Raveen Hanwella

**Affiliations:** 1 Department of Clinical Medicine, Faculty of Medicine, University of Colombo, Colombo, Sri Lanka; 2 Department of Psychological Medicine, Faculty of Medicine, University of Colombo, Colombo, Sri Lanka

## Abstract

**Background:**

Several studies have shown that long-term lithium use is associated with a subtle decline in estimated glomerular filtration rate. This study compared mean estimated glomerular filtration rates (eGFR) in patients on long term lithium, against matched controls.

**Methods:**

Patients with bipolar affective disorder, who are on lithium (for at least a year), were compared against controls that were matched (1:1) for age, gender and presence or absence of diabetes or hypertension. The eGFR was calculated from creatinine values according to the ‘modification of diet in renal disease study’ (MDRD) formula and was compared between cases and controls. A meta-analysis was performed to compare our findings with similar studies in literature.

**Results:**

Forty seven patients met the inclusion criteria. They were matched with 47 controls. The eGFR values of lithium users were significantly lower (p = 0.04) compared to controls. This difference persisted between the subgroup of lithium users without comorbidities (diabetes and hypertension) and their controls but disappeared for lithium users with comorbidities and their controls. Nonetheless, lithium users had lower eGFR values in both subgroups. A meta-analysis of 9 studies showed a significant lowering in the glomerular filtration rate in lithium users compared to controls [mean difference -10.3 ml/min (95% confidence interval: -15.13 to -5.55, p < 0.0001)].

**Conclusions:**

Lithium causes a subtle decline in glomerular filtration rate; renal function needs to be monitored in patients on lithium treatment.

## Background

Lithium is an effective and inexpensive drug that has been used for years to treat bipolar affective disorder. It is a monovalent cation that is well absorbed by the gastrointestinal system and completely excreted by the kidneys. Lithium is freely filtered in the glomeruli and 90% of the filtered load is reabsorbed in the proximal tubule [1]. Renal dysfunction caused by lithium is termed lithium nephropathy, and can manifest in many ways [2]. Firstly, lithium may induce a transient natriuresis by antagonizing aldosterone. Secondly, it can cause nephrogenic diabetes insipidus which is the most common renal ill-effect of lithium. Thirdly, lithium toxicity may predispose to acute kidney injury (due to pre-renal failure secondary to polyuria or lithium induced neuroleptic malignant syndrome) [3]. Lithium is also known to cause a distal renal tubular acidification defect [2,4].

However, whether lithium plays a contributory role to the development of chronic kidney disease (CKD) is unclear. Traditionally, lithium has not been considered as a cause of CKD. Recent findings have challenged that view. One study showed that while a majority (85%) of patients on long term lithium had normal estimated glomerular filtration rates (eGFR), 15% had reduced eGFRs [5]. Another study assessed the eGFR values of two cohorts of patients; a group on long term lithium (up to 33 years) and a group of lithium naïve patients treated with other mood stabilizers. Patients on long term lithium had significantly lower eGFR values (corrected for age and gender) [6]. These observations raise the possibility of lithium contributing to a decline in GFR which may eventually lead to CKD. In view of this new evidence, and considering the large patient population currently on lithium, investigating whether long term lithium use causes a reduction in eGFR is timely. The objectives of this study were to compare the eGFR values of patients on long term lithium with controls matched for age, gender and comorbidities (diabetes and hypertension).

## Methods

### Study design and sample selection

This was a comparative cross sectional study carried out by the University Medical Unit and the University Psychiatry Unit of the National Hospital of Sri Lanka. All consenting adult patients (18 years and above) who were on lithium and followed up at the University Psychiatry Unit as inpatients or outpatients were included in the study, and their data was obtained from the clinical records. The latest and previous creatinine values of patients were recorded, and eGFR values were calculated using the MDRD (Modification of Diet in Renal Disease) formula [7]. We also extracted data of patients with bipolar affective disorder who were not on lithium. These two groups were defined as below.

Group A: Patients with ICD-10 (International Classification of Diseases) clinical diagnosis of bipolar affective disorder who had been treated with lithium for a minimum period of one year. This group was further divided into two subgroups depending on whether the patients had diabetes or hypertension as a co-morbidity (subgroup A1: lithium users without diabetes or hypertension, subgroup A2: lithium users with diabetes, hypertension or both).

Group B: Patients with ICD-10 clinical diagnosis of bipolar affective disorder but not treated with lithium within the last 12 months. These patients were on treatment with other mood stabilizers.

### Matching

Matching was done on a 1:1 basis for patients in subgroups A1 and A2 (group B was too small to carry out any comparisons). Patients from the University Medical Unit, who were admitted to wards or followed up in clinics due to a non-renal illness or for evaluation purposes (and therefore had a serum creatinine value recorded) were recruited as controls. The clinics of the University Medical Unit have a population of approximately 1500 patients followed up as outpatients. The selection of a control was done by checking each registered patient on a randomly selected clinic date according to the matching criteria detailed below.

Controls for subgroup A1: Patients without a) a psychiatric condition (and not on lithium), b) diabetes or hypertension and c) any other comorbidity known to effect the eGFR were matched to each of the A1 patients with respect to age and gender on a 1:1 basis.

Controls for subgroup A2: Patients without a psychiatric condition (and not on lithium) but having either a) diabetes, b) hypertension or c) both were matched for each of the group A2 patients (1:1 match) with regard to the age, gender and the type of co morbidity. For example, for a lithium-using 56 year old male with diabetes and hypertension, a non lithium-using 56 year old male with diabetes and hypertension was selected as the control.

### Data extraction

Data pertaining to following aspects were collected from the recruits using a pre-tested interviewer administered data sheet: demography, past medical history, past psychiatric history, treatment history of lithium, complications of lithium therapy and serial creatinine values while on lithium.

The following patient categories were excluded from this study: a) patients on lithium diagnosed with renal disease or CKD due to another aetiology, b) non-consenting patients, c) patients with acute kidney injury, d) patients with an alternative cause for a high serum creatinine (e.g. myositis), e) patients with poor compliance with lithium and f) extremes of body mass index (18 < and > 30 kg/m^2^).

Ethical clearance for the study was granted by the Ethics Review Committee of the National Hospital of Sri Lanka.

### Statistical analysis

eGFR values were compared between the groups using mean differences. Significance of mean differences in eGFR between groups was calculated with the Independent t test. Statistical significance was set at p < 0.05. Descriptive statistics were summarized into proportions and averages based on the scales of measurement. SPSS statistical software (version 15) was used for data analysis. Linear regression was used to correlate two continuous variables.

### Meta-analysis

A recent meta-analysis has compared the eGFR values between lithium users and controls from studies published up to 2010 [8]. We built on that meta-analysis. We searched EMBASE, SCOPUS and PUBMED for publications between January 2011 – October 2013 which had the keywords “Lithium” and “glomerular filtration rate” or “GFR” or “eGFR” in the abstract. This time period was not covered by the previous meta-analysis. Three authors independently carried out the search and the data was filtered using Endnote X4 software (Thomson Reuters, Carlsbad, CA 92011, USA). Relevant studies published after 2010, and the data from our study were added to the data of studies identified in the previously published review and a new independent meta-analysis was carried out using the Revman (version 5) software [9].

## Results

### Results of the study

Forty seven patients were recruited to group A and six patients to group B. Of the group A patients, 24 belonged to subgroup A1 and 23 to subgroup A2. Of the patients in subgroup A2, 11 had diabetes, six had hypertension and six patients had both co-morbidities. The characteristics of group A and its controls are given in Table 1. Since the numbers were small in group B, they were used for an overall comparison of eGFR only (Table 2).

**Table 1:** Characteristics of cases and controls in the study

**Variable**	**Patients on Lithium without hypertension or diabetes (Group A1)**	**Patients on Lithium with hypertension and diabetes (Group A2)**	**Controls for Group A1**	**Controls for Group A2**
	**(n-24)**	**(n-23)**	**(n- 24)**	**(n-23)**
	**Mean ± SD**			
Mean age	42.68 ± 13.3	57.68 ± 9.3	42.66 ± 14.1	57.61 ± 8.9
Mean duration of diabetes (in years)	N/A	3.22 ± 3.3	N/A	6.77 ± 3.0
Mean duration of hypertension (in years)	N/A	5.42 ± 5.1	N/A	7.85 ± 4.8
Range of dose of lithium (mg)	250 - 1500	250 - 1000	N/A	N/A
Mean duration of lithium use (in years)	9.04 ± 5.9	11.5 ± 9.3	N/A	N/A

*N/A*: not applicable.

**Table 2:** Mean estimated glomerular filtration rates (eGFR) of different subgroups of lithium users compared to respective control groups

**Group**	**Mean (ml/min/1.73 m**^**2**^**) ± SD**	**T statistic**
Patients on lithium without hypertension or diabetes (Group A1, n - 24)	83.12 ± 27.4	2.07*
Controls for group A1 (n- 24)	97.01 ± 18.4	
Patients on lithium with hypertension and diabetes (group A2, n - 23)	75.85 ± 15.1	0.97
Controls for group A2 (n – 23)	81.27 ± 21.5	
Patients with bipolar affective disorder who were not on lithium (n – 6)	98.58 ± 32.3	Not used for comparison

*p < 0.05.

Group A had a higher number of patients with hypothyroidism which is a known complication of long term lithium therapy (seven patients among lithium users vs. one patient in the control group). The mean duration of diabetes and hypertension were greater among controls than the cases. Comparing the two subgroups (A1, A2) of lithium users, those with comorbidities (A2) were older and had been on lithium for a longer duration (Table 1). None of the lithium users had a recorded history of any other lithium related renal complication such as diabetes insipidus, acute kidney injury or renal tubular acidosis.

The patients on lithium (Group A, n = 47) had a mean eGFR value of 79.71 ml/min/1.73 m^2^ (SD ± 22.6, median: 79.9, interquartile range: 70.5 – 94.3). The age and gender matched controls (n =47) had a mean eGFR value of 89.31 ml/min/1.73 m^2^(SD ± 21.3, median: 90.6, interquartile range: 77.2 – 105.7). This difference was statistically significant (t- 2.12, df – 92, p = 0.04). Serial data on creatinine values were available for only 34 patients among the lithium users and the mean rate of decline of eGFR per year in that subgroup was -2.7 ml/min/1.73 m^2^ (SD ± 9.8). The comparisons of mean eGFR of the sub groups are summarized in Table 2. Notably, impairment in eGFR remains statistically significant for the comparison between subgroup A1 and their controls. Correlation of eGFR values with duration of lithium therapy by linear regression (controlling for age and gender) showed a negative trend which was not statistically significant (beta = -0.07, B = -0.16, p = 0.66).

### Results of the meta-analysis

**Figure 1:**
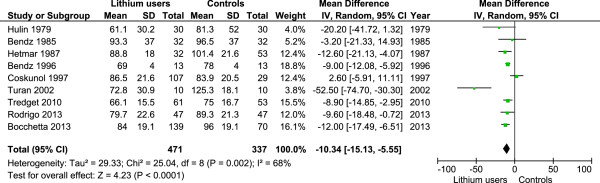
Forest plot for the comparison of glomerular filtration rates of lithium taking patients and controls.

The previously published review by McKnight et al. [8] had considered six studies in their paper [10-15]. We traced the original papers of these studies and re-entered the data for a new meta-analysis. Our search for the period from 2011 January to 2013 October recovered only one study with usable data [6]. There was another suitable study published in 2010 which had not been included in the earlier meta-analysis [16]. These data plus the results of our study were considered for the new meta-analysis. For five out of six studies of the earlier meta-analysis, the GFR values extracted by us were the same. However, in the remaining study (by Turan et al. [15]) we noted that the original authors had considered three groups of patients namely; lithium naïve patients, short term lithium users (less than three years) and long term lithium users (greater than 3 years). It seems the authors of the review had opted to include the GFR of short term lithium users for comparison. We felt that given the duration of lithium use in our study and other studies included in the meta-analysis, it was more appropriate to take the GFR values of the long term user group. This changed the direction of overall effect for that study compared to what was published in the previous meta-analysis [15] (See below). The results of our meta-analysis confirmed that lithium users had significantly lower GFR values compared to non-using controls; (9 studies, mean difference -10.3 ml/min, 95% CI: -15.13 to -5.55, 471 cases, 337 controls, p < 0.0001) (Figure 1). A summary of studies included in the meta-analysis is given in Table 3.

**Table 3:** Characteristics of studies included in the meta-analysis

**Study**	**Methods**	**Results (values are given as mean ± SD)**	**Comments**
Hullin et al. [10]	Comparative study	Mean duration of lithium treatment; 8.3 ± 2.8 years	GFR assessed by creatinine clearance
	Lithium group; 30 patients	GFR of lithium group; 61.1 ± 30.2 ml/min	
	Control group; age and gender matched 30 psychiatric patients not on lithium	GFR of controls; 81.3 ± 52.0 ml/min	
Bendz et al. [11]	Comparative study	Mean duration of lithium treatment; 5.7 ± 2.7 years	GFR assessed by endogenous creatinine clearance was taken for meta-analysis
	lithium group; 32 patients	GFR of lithium group; 90 ml/min	
	Control group; 32 age and gender matched psychiatric patients not on lithium	GFR of controls; 94 ml/min	
Hetmar et al. [13]	Comparative study	Mean duration of lithium treatment; 10 years	GFR assessed by endogenous creatinine clearance was taken for meta-analysis
	lithium group; 32 patients	GFR of lithium group; 88.8 ± 18.0 ml/min	When corrected for the age difference, there was no significant difference between the GFR of two groups
	Control group; 53 non matched patients with affective disorders not on lithium	GFR of controls; 101.4 ± 21.6 ml/min	
Bendz et al. [12]	Comparative study	Mean duration of lithium treatment; 18 years	GFR assessed by endogenous creatinine clearance
	lithium group; 13 patients	GFR of lithium group; 69 ± 4 ml/min	
	Control group; 13 age and gender matched psychiatric patients not on lithium	GFR of controls; 78 ± 4 ml/min	
Coskunol et al. [14]	Comparative study	Mean duration of lithium treatment; 4.5 ± 3.9 years	This is the only study that showed the lithium group to have a higher GFR value. However, the numbers in control group were few and they were not matched for cases.
	lithium group; 107 patients	GFR of lithium group; 86.5 ± 21.6 ml/min	GFR assessed by endogenous creatinine clearance
	Control group; 29 psychiatric patients not on lithium and without bipolar affective disorder	GFR of controls; 83.9 ± 20.5 ml/min	
Turan et al. [15]	Comparative study	Mean duration of Lithium treatment (for long term users); 6.6 ± 2.0 years	GFR assessed by endogenous creatinine clearance
	Lithium group; 20 patients (10 as short term users and 10 as long term users)	GFR of long term lithium group; 72.8 ± 30.9 ml/min	Considering the average duration of lithium use in other studies of the meta-analysis, long term lithium users were the appropriate group to include in the analysis in our opinion.
	Long term users had a duration of use more than 3 years	GFR of controls; 125.3 ± 18.1 ml/min	(please also see the comments in discussion)
	Control group; 10 lithium naïve unmatched patients with bipolar affective disorder		
Tredget et al. [16]	Comparative study	Mean duration of Lithium treatment; 15.6 ± 6.4 years	GFR was estimated with the modification of diet in renal disease (MDRD) study formula using the serum creatinine value and other parameters
	Lithium group; 61 patients	eGFR of long term lithium group; 66.1 ± 15.5 ml/min	
	Control group; 53 non lithium using patients with affective disorders	eGFR of controls; 75 ± 16.7 ml/min	
Bocchetta et al. [6]	Comparative study	Duration of lithium treatment; range – 1 to 33 years (mean duration not given), majority were users for more than 10 years according to a figure quoted in article	GFR was estimated with the modification of diet in renal disease (MDRD) study formula using the serum creatinine value and other parameters
	Lithium group; 139 patients with bipolar affective disorder	Mean difference of eGFR between cases and controls were; -12 ml/min (95% CI; -17.5 to 6.5 ml/min)	
	Control group; non matched 70 patients with affective disorders not on lithium, regression analysis used to control for confounding factors		
Rodrigo et al. (This study)	Comparative study	Mean duration of lithium treatment (for long term users); 10.1 ± 7.6 years	GFR was estimated with the modification of diet in renal disease (MDRD) study formula using the serum creatinine value and other parameters
	lithium group; 47 patients with bipolar affective disorder	eGFR of lithium group; 79.7 ± 22.6 ml/min	
	Control group; 47 people without a psychiatric illness matched for age, gender and comorbidities (diabetes and hypertension)	eGFR of controls; 89.3 ± 21.3 ml/min	

## Discussion

Many of the earlier comparative studies on the effect of lithium on eGFR/GFR have only controlled for the traditional variables of age and gender, and have not considered the effect of common comorbidities such as diabetes and hypertension. These were taken in to account in the more recently published study by Bocchetta et al. [6] but their adjustments for these confounders were done by means of a regression analysis. In this analysis age and gender showed a significant effect on eGFR (as expected) in addition to duration of lithium treatment. However, smoking, diabetes, dyslipidaemia and hypertension were not shown to have a significant effect on eGFR values. In our study, we attempted to control for some of these confounding factors when matching cases and controls. The controls were matched with regard to age, gender and the commonest comorbidities known to effect eGFR on the long term; diabetes and hypertension. This design of prospectively eliminating bias (rather than with a regression analysis) increased the validity of results.

Some studies quoted in the meta-analysis have classified patients into stages of CKD based on the eGFR [6,16]. However we did not classify our patients into eGFR categories meant for chronic kidney disease as this classification has opened up a debate about classifying apparently “healthy” people with eGFR values above 60 as having CKD stage II [17]. Furthermore, albuminuria is also now taken in to consideration in determining CKD risk and this data was not available for many studies including ours. Therefore, we restricted ourselves to a comparison of mean eGFR values of cases and controls only.

Overall, it was noted that patients on lithium (either with or without comorbidities) had lower eGFR values compared to their respective control groups. Our study demonstrates that long term lithium use may be an independent risk factor for a decline in eGFR even after controlling for age and gender related changes. When patients have comorbidities such as diabetes and hypertension, chronic lithium use may add on to the burden of declining eGFR but it is difficult to discern the effect of lithium from the effects of these illnesses. We only controlled the groups for the presence or absence of diabetes/hypertension but not for the duration or degree of control of these comorbidities. It is noted that patients in the control group had a longer mean duration of diabetes and hypertension (hence higher propensity for resultant renal impairment) compared to the lithium users with these complications. That might have prevented us from observing a significant difference in the eGFR in this subgroup (A2) and their controls.

One of the first studies that suggested a role of long term lithium use precipitating end stage kidney disease was a large population based study in Sweden. The investigators demonstrated that lithium users had a six-fold greater risk of needing renal replacement therapy compared with the general population [18]. However, the authors noted that end-stage kidney disease attributable to lithium is uncommon (only 18 patients were detected out of 3369 lithium treated patients). The mean duration of lithium use among these patients was 23 years. In our study, none of the lithium using patients had an eGFR value in the end stage kidney disease range, but the mean duration of lithium use in our sample was less than 10 years. In the previous study, the prevalence of CKD (excluding those with end stage disease) in the lithium treated general population was around 1.2% (CKD was defined as a serum creatinine value above 150 μmol/l).

The previously mentioned meta-analysis on lithium induced complications and toxicity concluded that on average, lithium use was associated with a – 6.22 ml/min/1.73 m^2^ lower GFR compared to controls [8]. This data came from six comparative studies. Two of them have demonstrated a significant drop in eGFR in lithium users [12,13] while two others have reported a non-significant decline in eGFR [10,11]. The overall effect was towards a reduction in eGFR, but this was not statistically significant. An important reason for this non-significant result was the study by Turan et al. [15] which had reported a paradoxical rise in GFR in lithium users. We interpreted the results of this study differently as mentioned above. This resulted in a change in the direction of effect for this study which was in keeping with the results of other studies, and the overall effect for the six studies in the original meta-analysis became significant. After adding the results of our study and the other two studies, the meta-analysis showed a significantly lower value of eGFR/GFR in chronic lithium users compared to non using controls.

The practice of liberal use of lithium as the drug of choice in bipolar affective disorder without proper monitoring of renal function has to be revisited in the light of these findings. However, it must be noted that the decline of eGFR/GFR with lithium use is subtle (although significant) and only a small proportion of lithium using patients may end up in end stage kidney disease. The exact pathology of lithium induced GFR change is still unknown and it should not be reduced to a simplistic view of longer duration of use causing renal impairment with time. Not all patients who are on long term therapy develop end stage disease, and our study and well as that by Tredget et al. [16] showed that duration of lithium use only had a non-significant negative association with eGFR. It is highly likely that other factors (individual and environmental) may be at play and future research should be aimed at elucidating the relationships between potential secondary contributing factors.

### Limitations

The numbers in our lithium using patient sample was limited by the fact that we excluded patients with poor compliance (patient reported compliance). We did not measure the serum lithium level of our recruited patients as a single current lithium level does not reflect compliance over many years. Unfortunately, facilities to measure serum lithium is not regularly available to patients free of charge and therefore such measurements had not been done for us to have an idea about patient compliance over time. The state-run clinics like ours provide free health care to patients, but investigations like serum lithium level are not available all the time. Most patients seeking health care in state-run cannot afford to get serum lithium levels tested from private sector laboratories. Several patients in the lithium-using category did not have periodic serial serum creatinine values in clinic records. Such patients were excluded from the analysis of the rate of decline in eGFR. Current guidelines suggest that eGFR alone is an inadequate measure to define at risk groups for CKD, and albuminuria also has to be taken in to account [19]. This measure was not compared between cases and controls in our study. Therefore, we have not classified patients and controls according to CKD risk categories or stages.

## Conclusions

This study showed that patients on long-term lithium had lower eGFR values that were statistically significant compared to age and gender matched controls. When patients with long standing diabetes and hypertension (confounders) were excluded from the analysis, the statistical significance persisted, indicating that chronic lithium use may in fact be the cause for the lower eGFR. Comparison of a subgroup of lithium users with diabetes and hypertension with respective controls showed a non-significant decline in eGFR in lithium users. A new meta-analysis carried out by us based on a meta-analysis published in 2012 (of studies published up to 2010) concluded that lithium causes a significant decline in eGFR/GFR. Despite the decline of GFR, end stage kidney disease associated with lithium seems to be uncommon. Nonetheless, in view of these findings, we suggest that psychiatrists remain vigilant and monitor the renal function of patients on long term lithium.

## Competing interests

The authors declare that they have no competing interests.

## Authors’ contributions

CR and VS conceptualized the study. CR, VS, SR and RH planned the design and the proposal. RH and VH were responsible for clinical care of patients. CR, NS and RG collected data and maintained the database. CR carried out the initial study analysis, meta-analysis and wrote the first draft. SR, VS and RH reviewed the statistics. All authors contributed to and approved the final manuscript.

## Authors’ information

CR (MBBS, MD) is lecturer in Medicine and NS (MBBS) and RG(MBBS) are research assistants attached to the Department of Clinical Medicine, Faculty of Medicine, University of Colombo. SR (MBBS, MD, FRCP, FRCPE, FACP, FCCP) is Professor in Medicine attached to the same department. RH (MBBS, MD, MRCPsych) and VS (MBBS, MD) are consultant psychiatrists and Professors attached to the Department of Psychological Medicine, Faculty of Medicine, University of Colombo.

## Pre-publication history

The pre-publication history for this paper can be accessed here:

http://www.biomedcentral.com/1471-244X/14/4/prepub
